# Histogram analysis of ADC in rectal cancer: associations with different histopathological findings including expression of EGFR, Hif1-alpha, VEGF, p53, PD1, and KI 67. A preliminary study

**DOI:** 10.18632/oncotarget.24905

**Published:** 2018-04-06

**Authors:** Hans Jonas Meyer, Annekathrin Höhn, Alexey Surov

**Affiliations:** ^1^ Department of Diagnostic and Interventional Radiology, University Hospital of Leipzig, 04103 Leipzig, Germany; ^2^ Department of Pathology University Hospital of Leipzig, 04103 Leipzig, Germany

**Keywords:** rectal cancer, DWI, ADC, histogram analysis

## Abstract

Functional imaging modalities like Diffusion-weighted imaging are increasingly used to predict tumor behavior like cellularity and vascularity in different tumors. Histogram analysis is an emergent imaging analysis, in which every voxel is used to obtain a histogram and therefore statistically information about tumors can be provided. The purpose of this study was to elucidate possible associations between ADC histogram parameters and several immunhistochemical features in rectal cancer. Overall, 11 patients with histologically proven rectal cancer were included into the study. There were 2 (18.18%) females and 9 males with a mean age of 67.1 years. KI 67-index, expression of p53, EGFR, VEGF, and Hif1-alpha were semiautomatically estimated. The tumors were divided into PD1-positive and PD1-negative lesions. ADC histogram analysis was performed as a whole lesion measurement using an in-house matlab application.

Spearman's correlation analysis revealed a strong correlation between EGFR expression and ADCmax (p=0.72, P=0.02). None of the vascular parameters (VEGF, Hif1-alpha) correlated with ADC parameters. Kurtosis and skewness correlated inversely with p53 expression (p=-0.64, P=0.03 and p=-0.81, P=0.002, respectively). ADCmedian and ADCmode correlated with Ki67 (p=-0.62, P=0.04 and p=-0.65, P=0.03, respectively). PD1-positive tumors showed statistically significant lower ADCmax values in comparison to PD1-negative tumors, 1.93 ± 0.36 vs 2.32 ± 0.47×10^−3^mm^2^/s, p=0.04.

Several associations were identified between histogram parameter derived from ADC maps and EGFR, KI 67 and p53 expression in rectal cancer. Furthermore, ADCmax was different between PD1 positive and PD1 negative tumors indicating an important role of ADC parameters for possible future treatment prediction.

## INTRODUCTION

Colorectal cancer is the fourth most common malignancy in the united states [1]. One third of these cases are located in the rectum.

Magnetic resonance imaging (MRI) plays an important role in local staging of rectal cancer [2]. Beside correct staging, MRI can also provide additional information regarding tumor microstructure. So far, diffusion weighted imaging (DWI) by means of apparent diffusion coefficient (ADC) has been described as a diagnostic tool for estimation of tissue composition and behavior of several tumors [3]. It has been shown that ADC is associated with cellularity in different malignant and benign lesions [3]. For rectal cancer, it has been shown that ADC is associated with tumor cell count and Ki67 [4, 5]. Moreover, some studies identified correlations between ADC and microvessel density parameters [6]. Of further clinical importance is that ADC values are also significantly different between several tumor stages [7–9]. Additionally, ADC can predict treatment response to radiochemotherapy [7–9].

A promising imaging analysis technique is histogram analysis, which issues every voxel of a region of interest (ROI) into a histogram and therefore, information regarding tumor homogeneity/heterogeneity can be obtained [10]. Histogram analysis includes several parameters like percentiles, mode, median, and second order statistical parameters, namely kurtosis, skewness and entropy [10]. Especially entropy, a marker of the heterogeneity of the histogram might reflect tumor heterogeneity, as it was exemplarily shown in cervical cancer [11]. Presumably, using this approach, more associations between histopathology and ADC values can be identified, which might not be found with conventional ROI based analysis alone [10].

Several different immunohistochemical markers haven been investigated in rectal cancer [12]. They can aid for prognosis prediction, treatment success of radiotherapy and to further stratify patient groups [12, 13]. Ki67 is a widely used marker for proliferation estimation and might be associated with tumor behavior, although the published results are inconclusive [13]. A further biomarker, namely P53, is the most investigated tumor suppressor antigen and it is mutated in almost all tumor entities [14]. Epidermal growth factor receptor (EGFR), a tumor oncogene is involved in the regulation of many cellular responses, including cell proliferation, apoptosis, and cellular differentiation [15]. According to the literature, it is widely expressed in several tumor entities [15].

Hif-1alpha is a protein expressed during hypoxic states of the cell to stimulate angiogenesis and, thus, to maintain tumor growth [16]. In rectal cancer, it is associated with prognosis and response for treatment with anti-angiogenesis drugs [16].

Recently, programmed cell death protein (PD-1) was identified to be a promising aim for treatment, called check point inhibitors. This treatment was especially investigated for malignant melanoma und non-small cell lung cancer with very promising results [17]. Previous studies indicated that PD-1 expression is also present in rectal cancer, which might lead to a new treatment regime in the future [17].

Previously, only few studies investigated possible associations between ADC values and histopathology findings like cellularity, KI 67 and VEGF [4, 6, 18, 19], but none of them used the histogram approach. Presumably, ADC values might not only be able to reflect cellularity in rectal cancer but also other histopathological markers.

Therefore, the purpose of this present study was to investigate possible associations of ADC values derived from histogram analysis with several biomarkers in rectal cancer.

## RESULTS

Spearman's correlation analysis revealed a strong correlation between EGFR expression and ADCmax (p=0.72, P=0.02) (Table 1). Kurtosis (p=-0.64, P=0.03) and skewness (p=-0.81, P=0.002) correlated inversely with p53 expression. Furthermore, ADCmedian and ADCmode correlated with Ki67 (p=-0.62, P=0.04 and p=-0.65, P=0.03, respectively). Finally, P10 and P25 tended to correlate with KI 67.

**Table 1:** Correlation analysis between ADC histogram parameters and histopathological findings

Parameter	EGFR	Hif1-alpha	VEGF	p53	KI 67
ADCmean	p=0.48, P=0.15	p=0.29, P=0.39	p=0.26, P=0.44	p=0.26, P=0.45	p=-0.56, P=0.07
ADCmin	p=0.43, P=0.22	p=0.49, P=0.12	p=-0.10, P=0.79	p=-0.12, P=0.73	p=-0.49, P=0.13
ADCmax	**p=0.72, P=0.02**	p=0.21, P=0.54	p=0.42, P=0.20	p=-0.12, P=0.71	p=-0.08, P=0.81
ADCp10	p=0.52, P=0.13	p=0.34, P=0.31	p=-0.05, P=0.87	p=-0.26, P=0.43	p=-0.57, P=0.06
ADCp25	p=0.48, P=0.17	p=0.25, P=0.46	p=0.17, P=0.61	P=0.22, P=0.51	p=-0.60, P=0.05
ADCp75	p=0.41, P=0.25	P=0.25, P=0.45	p=0.33, P=0.33	p=0.26, P=0.43	p=-0.43, P=0.19
ADCp90	p=0.32, P=0.37	p=0.29, P=0.38	p=0.49, P=0.12	p=0.26, P=0.44	p=-0.35, P=0.15
ADCmedian	p=0.50, P=0.14	p=0.26, P=0.43	p=0.25, P=0.45	p=0.26, P=0.43	**p=-0.62, P=0.04**
ADCmode	p=0.39, P=0.26	p=-0.09, P=0.79	p=0.12, P=0.72	p=0.56, P=0.07	**p=-0.65, P=0.03**
Kurtosis	p=0.09, P=0.81	p=0.25, P=0.47	p=-0.15, P=0.65	**p=-0.64, P=0.03**	p=0.46, P=0.16
Skewness	P=-0.03, P=0.95	p=0.36, P=0.27	p=0.23, P=0.50	**p=-0.81, P=0.002**	p=0.33, P=0.32
Entropy	p=0.45, P=0.19	p=-0.14, P=0.69	p=-0.12, P=0.80	p=0.16, P=0.63	p=-0.15, P=0.65

Statistically significant correlations are highlighted in bold.

PD1-positive tumors showed statistically significant lower ADCmax values in comparison to PD1-negative tumors 1.93 ± 0.36 vs 2.32 ± 0.47×10^−3^ mm^2^/s, p=0.04 (Table 2).

**Table 2:** Differentiation between PD1 positive and PD1- negative tumors

Parameter	PD1- negative	PD1- positive	p-value
ADCmean	1.21 ±0.25	1.15 ±0.15	0.63
ADmin	0.77± 0.25	0.60± 0.24	0.50
ADCmax	2.32± 0.47	1.93 ±0.36	0.04
P10	0.87± 0.35	0.89 ±0.18	0.78
P25	1.03 ±0.29	1.01 ±0.15	0.98
P75	1.37 ±0.23	1.28 ±0.19	0.63
P90	1.51 ±0.21	1.41 ±0.20	0.38
ADCmedian	1.17 ±0.26	1.13 ±0.15	0.92
ADCmode	0.98 ±0.26	1.05 ±0.16	0.92
Kurtosis	4.20 ±1.03	3.61± 1.67	0.50
Skewness	0.89 ±0.31	0.46± 0.54	0.28
Entropy	2.83 ±0.54	2.87± 0.68	0.79

Statistically significant correlations are highlighted in bold.

## DISCUSSION

This present study identified significant associations between several histopathological features and histogram parameters derived from ADC maps in rectal cancer.

ADC values are widely acknowledged to be mainly influenced by cellularity [3]. When cell density is increasing, free diffusion of water molecules is hindered and, therefore, ADC value is lowered [3]. However, this association seems to be different in several tumors, as shown in recent meta-analyses [3, 20]. Furthermore, it was reported that other histopathological parameters such as extracellular matrix [21], tumor size, and cell membranes [22] can also influence free diffusion of water molecules and ADC values.

As another aspect, ADC was used for prognostic evaluation in several cancer entities [23–26]. According to De Felice et al., ADC was able to predict treatment response to radiochemotherapy in rectal cancer [23].

There is increasing evidence that ADC reflects KI67 index in several tumors [5]. In rectal cancer, a correlation coefficient of r=-0.42 was identified in a recent meta-analysis [5], with a range from r=-0.30 to −0.49 indicating only a moderate correlation in this tumor entity [4, 18, 19]. However, no histogram analysis was used to identify further associations with Ki67 index in these studies. In the present study, ADC median and ADCmode correlated inversely with KI 67 index, even higher than the previously reported correlation coefficients.

P53 is one of the most known tumor suppressor gene [27]. It plays an important role in the regulation of cell proliferation and apoptosis [27]. For rectal cancer, p53 is associated with survival [27]. Therefore, prediction of p53 expression by MRI might have a crucial benefit in clinical routine. Previously, several studies analyzed associations between ADC values and P53 expression in different tumors. For example, Heijmen et al. found no significant correlation between ADC and p53 in liver metastases from colorectal cancers [28], whereas Sevcenco et al. identified a significant correlation between these parameters in bladder carcinomas [29]. Moreover, Schob et al. identified a positive correlation between ADC entropy and p53 count in uterine cervical cancer [11]. However, no study to date analyzed possible association between ADC values and p53 expression in rectal cancer. In the present study, an inverse strong correlation between P53 expression and skewness was identified. This finding seems to be logical. In fact, skewness represents a measure of asymmetry of histogram distribution [10]. Negative skewness represents few low ADC values and a higher amount of high ADC values, whereas positive skewness represents a lot of low ADC values with a low amount of high ADC values [10]. Another parameter, namely kurtosis was also correlated with p53 expression but a bit weaker with a coefficient of −0.64. This parameter represents the peaknedness of histogram [10].

Regarding vascularity related parameters, only few studies have been published previously. Yet, the results indicated that there might be a link between vessel density and ADC values. For example, Bäuerle et al. identified a significant correlation between the true diffusion coefficient (D) and mean vessel density in 21 patients [6]. Furthermore, D correlated also with vascular area [6]. This finding is difficult to understand because for the calculation of D the perfusion related proportion of ADC is excluded and, therefore, hypothetically, no correlation should be identified. In another study, the perfusion fraction f, a perfusion parameter derived from low b-values, showed a positive correlation with mean vessel density [4]. Furthermore, Meng et al. identified weak inverse correlations between ADC values and VEGF expression (r=-0.29) and Hif1-alpha expression (r=-0.304) [18]. Contrary, in the present study no significant correlations between ADC histogram analysis parameters and expression of VEGF or Hif1-alpha were found. A possible explanation for this phenomenon might be the fact that the perfusion fraction derived from DWI might be better to reflect vascularity parameters than histogram analysis.

EGFR expression is known to activate a cascade of multiple signaling pathways that enable tumor growth process [30]. Furthermore, high EGFR expression was identified to be associated with more aggressive disease, advanced tumor stage and increased risk of metastases [30]. In addition, high EGFR expression can predict results of radiochemotherapy [30]. The present study showed that ADCmax might be used as a surrogate marker for EGFR expression. Overall, this is the first analysis regarding associations between ADC values and EGFR expression to date. However, the identified positive correlations cannot be easily ascertained. Presumably, higher EGFR expression leads to tumors with more aggressive nature and, thus, more densely packed cells with larger nuclei. These facts may result in an inverse correlation between these parameters. We hypothesize that due tumor necrosis the ADC might be higher and ADCmax might be sensitive to reflect this histopathological feature.

The benefit of ADCmax, as the voxel containing the highest ADC value and thus a marker for the highest diffusion in the tumor, is not well investigated to date. However, some studies indicated that ADCmax might also be an important ADC parameter reflecting a different aspect of tumor microstructure than the other parameters [31–33]. For example, in meningioma it was associated with aquaporin 4 expression, a water channel protein in cell membranes [31]. In another study, investigated ovarian cancer xenografts the maximum ADC was a good indicator of treatment-induced cell death and changes in the extracellular matrix [33]. Moreover, ADCmax was associated with FIGO stage and lymph node status in cervical cancer [32]. Clearly, further studies are required to elucidate the potential benefit of this parameter.

PD1 is a transmembrane glycoprotein of the IG-superfamily that functions as a T cell co-inhibitory receptor [34]. Recently, a lot of studies investigated the antibody against PD1 as a potential treatment target in various tumors, such as in non-small cell lung cancer and malignant melanoma with very promising results [34].

In the present study, we found that only ADCmax was significantly different between PD1-positive and PD1-negative tumors. Previously, no studies to date investigated associations between ADC values and PD1-expression. This finding might be of interest, when treatment regimes with antibodies against PD1 are used in rectal cancers in the future.

ADC histogram analysis was recently used to analyze rectal cancers in other studies [35, 36]. In the study by van Heeswijk et al., it could be shown that histogram derived parameters have an excellent interobserver agreement with intraclass correlation coefficients ranging between 0.80 and 0.98 [35]. This finding underlines the validity of this method and its possible implementation into clinical routine. Furthermore, they used a non-precise tumor delineation, which was faster with similar results by the measurement by an expert radiologist, indicating that it could be performed in a semiautomatically fashion with presumable also good interobserver agreement. This finding is also very important when considering the implementation of histogram analysis into clinical routine. Interestingly, the calculated ADC parameters in this mentioned study are quite good comparable with our calculated ADC parameters, although the software algorithm might differ [35]. Furthermore, Choi et al. showed that histogram derived parameters differ significantly before and after radiochemotherapy. Thereby, the ADC values tend to rise after therapy, indicating a consecutive decrease of cell density [36]. In addition, the 25^th^ percentile was the best parameter to predict pathological complete response with an area under the curve of 0.768 [36].

There are several limitations of this study to address. Firstly, it is a retrospective study with possible known bias. However, the histopathology and imaging were examined independently and blinded to each other. Secondly, and surely the most important one, is the small patient sample size. Thirdly, the investigated bioptic samples only represent a relatively small portion of the tumor, whereas the MRI was analyzed as a whole tumor measurement.

Clearly, further prospective studies are needed to overcome the limitations of this present study and to confirm these preliminary results.

In conclusion, this study identified associations between histogram parameter derived from ADC maps and EGFR, KI 67 and p53 expression in rectal cancer. Furthermore, ADCmax was different between PD1 positive and PD1 negative tumors indicating an important role in possible future treatment prediction.

## MATERIALS AND METHODS

This retrospective study was approved by the local ethic committee.

### Patient sample

We screened our radiology database for patient with rectal cancer, including DWI and available bioptic samples in the pathology department. Overall, 11 patients with histologically proven rectal cancer met the inclusion criteria (Table 3). There were 2 (18.18%) females and 9 male with a mean age of 67.1 years (range, 51-76 years) with different tumor stages.

**Table 3:** Patients and tumors included into the study

N	Age	Sex	T stage	N stage	M stage	Grade
1	74	m	3	1	0	1
2	60	m	3	1	1	1
3	69	m	3	0	0	1
4	69	m	3	0	1	1
5	71	m	3	1	0	1
6	61	m	3	0	1	1
7	51	f	3	1	0	1
8	71	m	3	1	0	1
9	63	m	4	2	0	2
10	73	m	2	0	0	2
11	76	f	2	1	0	2

### MRI

In all patients MRI of the pelvis was performed by using a 3.0 T device (MagnetomSkyra, Siemens, Erlangen, Germany). The imaging protocol included the following sequences: axial and sagittal T2 weighted (T2w) fat-supressed (fs) short tau inversion recovery (STIR) images, axial T2w turbo spin echo images, axial T1 weighted turbo spin echo (T1w TSE) images, and axial T1w TSE images with fat suppression after intravenous application of contrast medium (gadopentate dimeglumine, Magnevist, Bayer Schering Pharma, Leverkusen, Germany), in a dosis of 0.1 ml per kilogram of body weight. DWI was performed using a multi-slice single-shot echo-planar imaging (EPI) sequence with b values of 0 and 1000 s/mm^2^

All images were analyzed by one radiologist (A.S., 14 years radiological experience) on a PACS workstation (Centricity PACS, GE Medical Systems, Milwaukee, Wis., USA).

### Histogram analysis

DWI data was transferred into DICOM format and processed offline with a custom-made Matlab-based application (The Mathworks, Natick, MA, USA). The VOI was manually created on ADC maps drawing regions of interest (ROIs) along the margin of the tumor using all slices with tumor (whole lesion measurement). Within this ROI, the following parameters were calculated: mean (ADCmean), maximum (ADCmax), minimum (ADCmin), median (ADCmedian), mode (ADCmode), and the following percentiles: 10th (ADCp10), 25th (ADCp25), 75th (ADCp75), 90th (ADCp90), as well as the second order statistics skewness, kurtosis and entropy.

### Histopathological analysis

**Figure 1:**
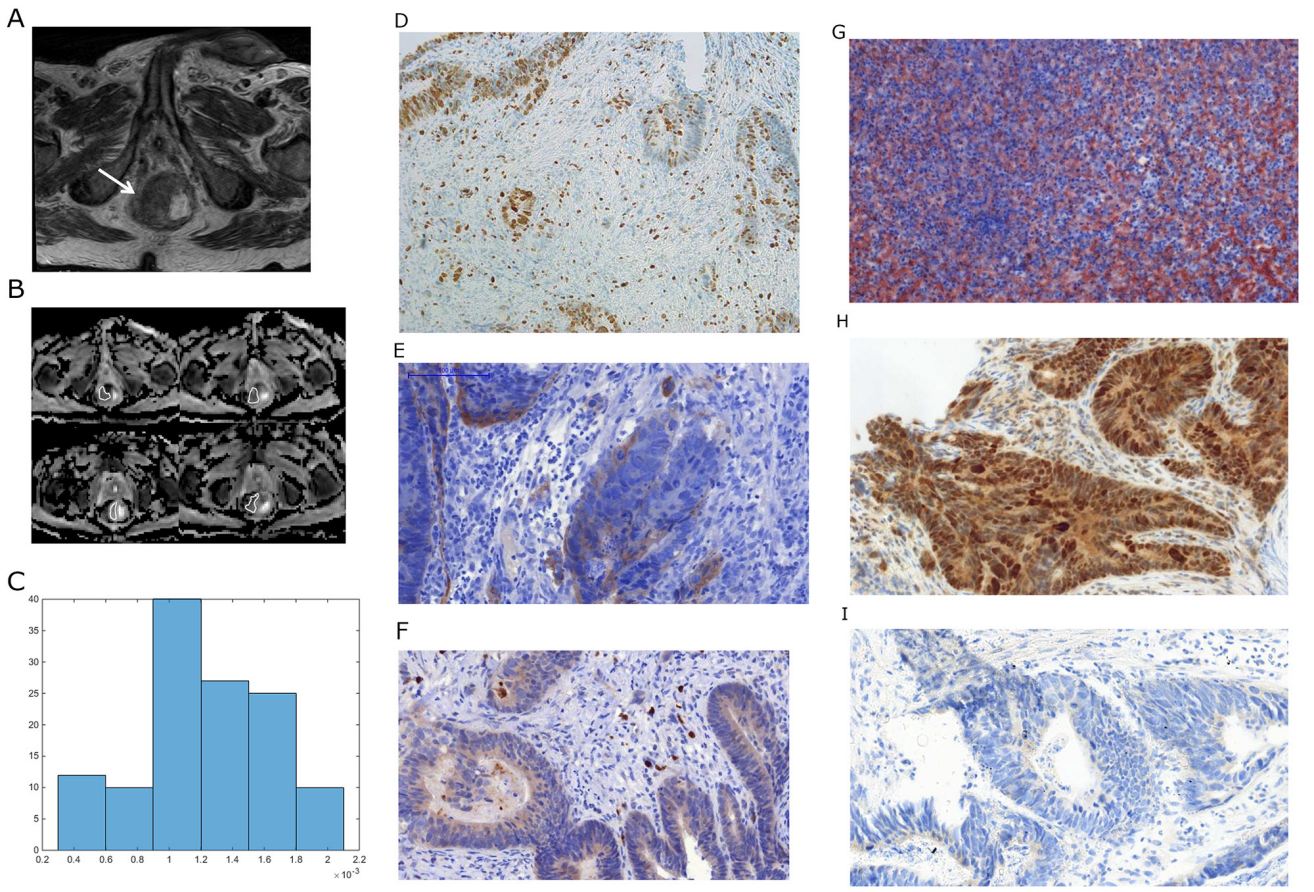
Imaging and histopathological findings in a patient with rectal cancer **(A)** T2 weighted image documenting a large rectal cancer (arrow). **(B)** ADC maps of the lesion with regions of interest (ROIs). **(C)** ADC histogram. The histogram analysis parameters (× 10^−3^ mm^2^s^−1^) are as follows: ADC_min_= 0.3, ADC_mean_= 1.23, ADC_max_= 1.96, P10 = 0.63, P25 = 0.99, P75 = 1.57, P90 = 1.77, median = 1.2, and mode = 1.04. Histogram-based characteristics are: kurtosis = 2.47, skewness = −0.26, and entropy = 2.38. **(D)** MIB 1 staining. KI 67 index is 23 %. **(E)** EGFR staining. Stained area is 8865 μm^2^. **(F)** Hif1alpha staining. Stained area is 1284μm^2^. **(G)** VEGF staining. Stained area is 30078 μm^2^. **(H)** P53 staining. Stained area is 78854 μm^2^. **(I)**. PD staining (weak positive).

The diagnosis of rectal cancer was confirmed histopathologically by endoscopic rectal biopsy in all cases. Representative tumor tissue slides from formalin-fixed paraffin-embedded tissue were processed after deparaffinization. The specimens were stained with several monoclonal antibodies, including MIB-1 (DakoCytomation, Glostrup, Denmark), VEGF(EMERGO Europe, Den Haag, The Netherlands); p53 (DakoCytomation, Glostrup, Denmark), EGFR (EMERGO Europe, Den Haag, The Netherlands) and Hif1-alpha (Biocare medical, Pacheco, USA) as well as PD-1 (Abcam, Cambridge, USA). All stained samples were digitalized by using a research microscope Jenalumar (Zeiss, Jena, Germany). Furthermore, the digital histopathological images were transferred as uncompressed TIFF images to the free available ImageJ software (version 1.48v, NIH, Bethesda, MD). Proliferation index (KI 67) was calculated as percentage of stained nuclei on the MIB-1 stained specimens as reported previously. The area with the highest number of positive tumor nuclei was selected for the analysis (Figure 1). In every case, all histopathological parameters were estimated per two high power fields a 0.16 mm^2^. Immunhistochemical expression was semiautomatically estimated using ImageJ and overall stained areas were measured according to previous descriptions [30, 37]. Finally, PD1- expression was estimated in PD1- positive and PD1-negative manually by stained cancer cells [17].

### Statistical analysis

GraphPad Prism (GraphPad Software, La Jolla, CA, USA) was used for statistical analysis and figure creation. Collected data were evaluated by means of descriptive statistics (absolute and relative frequencies). Categorical variables were expressed as percentages. Analyses of ADC parameters were performed by means of two sided Mann-Whitney-U-tests. P-values < 0.05 were taken to indicate statistical significance in all instances. Spearman's correlation coefficient was used to analyze the association between ADC and histological parameters.
